# Supplementary material for: Patients with Advanced or Metastasised Non-Small-Cell Lung Cancer with Viscum album L. Therapy in Addition to PD-1/PD-L1 Blockade: A Real-World Data Study
Source: Cancers (Basel). 2024 Apr 22;16(8):1609. doi: 10.3390/cancers16081609 (PMC11049173; doi:10.3390/cancers16081609)
Supplement: Supplementary file 1 [file cancers-16-01609-s001.zip › cancers-2924574-supplementary.pdf]

cancers

MDPI

Supplementary table S1. Molecular characteristics of EGFR mutations, n=13

	Total group (n=415)	CRTL (n=222)	COMB (n=193)	p-value
**EGFR mutation**				
**p.Leu747_Thr751delLeuArgGluAlaThrinsPro*, n (%)**	1 (0.2)	-	1 (0.5) ins, del	-
**p.Leu747_Pro753delinsSer*, n (%)**	1 (0.2)	-	1 (0.5) ins, del	-
**p.Asn771_Asn771delAsninsGlyThr**, n (%)**	1 (0.2)	1 (0.5) del	-	-
**p.Glu746_Ala750delGluLeuAr[1], n (%)**	1 (0.2)	1 (0.5) del, sens	-	-
**p.Glu746_Ala750delGluLeuArgGluAla[1], n (%)**	1 (0.2)	-	1 (0.5) del, sens	-
**p.Leu858Arg[1], n (%)**	6 (1.4)	3 (1.4) point, sens	3 (1.6) point, sens	0.86
**p.HisdupHis*, n (%)**	1 (0.2)	1 (0.5) dupl	-	-
**p.Ser768_Asp770dupSerValAsp**, n (%)**	1 (0.2)	-	1 (0.5) dup	-
**Total, n (%)**	13 (3.1)	6 (2.7)	7 (3.6)	0.50
**EGFR insertion mutations, n (%)**	2 (0.5)	-	2 (1.0)	-
**EGFR deletion mutations, n (%)**	5 (1.2)	2 (0.9)	3 (1.6)	0.54
**EGFR point mutations, n (%)**	6 (1.4)	3 (1.4)	3 (1.6)	0.86
**EGFR duplication mutation, n (%)**	2 (0.5)	1 (0.5)	1 (0.5)	0.92
**EGFR Mutation sensitive against TKI, n (%)**	8 (1.9)	4 (1.8)	4 (2.1)	0.84

*Molecular characteristics and types of EGFR-mutations; ins, insertion mutation; del, deletion mutation; point, point mutation; dupl, duplication mutation; sens, sensitive against tyrosine kinase inhibitors; TKIs, tyrosine kinase inhibitors; *no sensitivity against TKIs; **no sensitivity against TKIs of 1st, 2nd or 3rd generation; ***no information on TKI-sensitivity; [1] sensitive against TKI*

Supplementary table S2. Multivariate cox proportional analysis in advanced or metastasized NSCLC patients treated with PD-1/PD-L1 inhibitors

	aHR	(95% CI)	P-Value
**VA vs. non-VA**	0.451	0.282 - 0.720	0.0008***
**Age**	0.999	0.973 - 1.025	0.91
**Gender, female vs. male**	0.625	0.372 - 1.052	0.08.
**BMI 25 – 29.9 kg/m^2 vs. <25 kg/m^2**	0.614	0.369 - 1.022	0.06.
**BMI ≥30 kg/m^2 vs. <25 kg/m^2**	0.273	0.115 - 0.648	0.003 **
**ECOG status**	1.399	1.126 - 1.738	0.002 **
**Smoker, current/past vs. never**	1.342	0.622 - 2.895	0.45
**Comorbidities, yes vs. no**	0.418	0.246 - 0.710	0.0013 **
**Surgery, yes vs. non**	0.792	0.369 - 1.702	0.55
**Stage IV UICC vs. stage III UICC**	0.723	0.312 - 1.676	0.45
**Brain radiation vs. no brain radiation**	1.302	0.704 - 2.408	0.40
**PD-L1 TPS 1-10%**	0.766	0.418 - 1.403	0.38
**PD-L1 TPS 11-20%**	1.102	0.394 - 3.085	0.85
**PD-L1 TPS 21-30%**	0.822	0.341 - 1.979	0.66
**PD-L1 TPS 31-40%**	0.618	0.180 - 2.123	0.44
**PD-L1 TPS 41-50%**	6.001	1.301 - 27.684	0.022*
**PD-L1 TPS 51-60%**	0.570	0.212 - 1.536	0.27

**PD-L1 TPS 61-70%**	0.374	0.115 - 1.217	0.10
**PD-L1 TPS 71-80%**	0.491	0.145 - 1.668	0.25
**PD-L1 TPS 81-90%**	0.609	0.270 - 1.372	0.23
**PD-L1 TPS 91-100%**	0.718	0.149 - 3.448	0.68
**EGFR, positive**	1.068	0.477 - 2.391	0.87

*Adjusted multivariate cox proportional analysis of association of add-on VA with adjusted hazard of death in advanced or metastasized NSCLC patients treated with PD-1/PD-L1 inhibitors, n=162; aHR, adjusted hazard ratio; VA, Viscum album L.; BMI, body mass index; PD-L1, programmed death-ligand 1; TPS, tumour proportion score; EGFR, epidermal growth factor receptor *, p-value <0.05; **, p-value <0.01; ***, p-value <0.001*
